# Underestimating Children’s Self-reported Pain: Agree/Disagree?

**DOI:** 10.4274/TJAR.2024.241646

**Published:** 2025-10-14

**Authors:** Şefika Başoğlu, Özlem Selvi Can, Volkan Baytaş, Hakan Yılmaz, Fatma Nur Erkent

**Affiliations:** 1 Ankara University Faculty of Medicine Department of Anaesthesiology and Intensive Care, Ankara, Türkiye

**Keywords:** Child, pain measurement, paediatric anaesthesia, postoperative pain, proxy

## Abstract

**Objective:**

To compare postoperative pain after different surgical types and grades using the visual analogue scale (VAS) and numeric rating scale (NRS) evaluated by the patient, parent, and nurse.

**Methods:**

After approval from the local ethics committee and written informed consent from the patient and parent, a single-center, prospective, randomized study was designed. A total of 180 children with American Society of Anesthesiologists I-III physical status between the ages of 7-12 (n = 90) and 13-18 (n = 90) years were included in the study who underwent elective surgery at Ankara University Faculty of Medicine Hospitals between January and December 2022. Pain was assessed postoperatively at 2 hours using two pain scales. Patients who underwent mini-intermediate or major surgery were evaluated separately.

**Results:**

Four children from each age group were excluded from the study due to insufficient data recording, and data from 172 children were analyzed. Including all age groups and surgical grades, all children had excellent agreement with the parent [VAS/NRS: intraclass correlation coefficient (ICC)= 0.903/ICC= 0.900] and good agreement was found between the child and nurse (VAS/NRS: ICC= 0.852/ ICC= 0.842). For the VAS and NRS, when parent and nurse compliance scores were compared, no significant difference was observed between the two scores. For VAS and NRS, fathers were found to be better at predicting pain for children than mothers.

**Conclusion:**

Self-reported pain is the gold standard for pain evaluation. The parents assessed pain scores that were similar to those of their children using NRS and VAS. Nurses underestimated a child’s pain with both scores.

**Main Points**

• Pain is a subjective experience, and self-reporting is the gold standard for assessment and treatment.

• Furthermore, a child’s self-report on pain must be used whenever possible to guide pain management. Sometimes, it is not possible (due to developmental, linguistic, and cognitive limitations) for parents’ or nurses’ evaluations to be vital to managing pain therapy.

• Children’s self-pain reports are similar to those of their parents and are more reliable than nurses’ evaluations.

• In this study, the parents evaluated the children’s postoperative pain similarly using the visual analogue scale and numeric rating scale. Nurses underestimated children’s pain with both pain scores.

## Introduction

The International Association for the Study of Pain defines pain as “an unpleasant sensory and emotional experience associated with actual or potential tissue damage or threat”.^1^ It is every patient’s right to receive relief from their suffering and pain. The manner in which children express pain varies depending on their age and developmental stage. Because children sometimes have limited experience reporting pain, their inability to verbally express pain or feelings sometimes makes pain assessment difficult in children.^2^

Historically, assessing and reporting pain has been the gold standard for effective pain assessment.^3^ Paediatric patients comprise a broad population ranging from newborns to 18 years of age. Different pain assessment tools are available to evaluate pain types according to age in this large population.^2, 3, 4^

There are only a limited number of studies with different results regarding parents’ and nurses’ evaluations of children’s pain. Different results emerged from studies in which parents or medical professionals assessed pain in children. In some of these studies, it has been found that parents and doctors/nurses sometimes evaluate pain similarly to children but often give lower or higher scores than it is.^5, 6, 7, 8, 9, 10, 11, 12, 13^ Given the limited number of studies on this topic within our country, we undertook this research to address this gap. Due to the different ages of paediatric patients and difficulties they experience in expressing themselves, pain treatment in children should be evaluated and managed by an observer. In practice, a nurse in the hospital, as well as parents and other caregivers at home, perform this evaluation after discharge.^2^ It has been found that parents and nurses often underrate or overestimate pain.^8, 14^ There are risks in overestimating pain and unnecessary treatment, as well as in underestimating pain and inadequate treatment. Parents’ false beliefs that medications should only be given if their child has unbearable pain, their fear that their child may become addicted, or their belief that their child expresses pain to attract attention may lead to inadequate pain treatment.^15^

In postoperative pain management guidelines, some pain assessment tools have been validated for their precision in detecting the presence of pain and determining its severity. The visual analogue scale (VAS) and numeric rating scale (NRS) are commonly used one-dimensional pain scales and are widely used in children older than 4 years for self-reporting or assessment of their proxies in pain.^2, 4, 16^

Our study was planned to evaluate pain at the 2^nd^ postoperative hour first using the VAS and then using the NRS 11 by the child, parent, and nurse separately. Our primary aim was to determine which parents or nurses would perform the closest pain assessment for the child. This study also aimed to reveal whether the parent and nurse had differences in VAS or NRS pain scores when estimating the child’s pain most closely. Our secondary aims are to identify factors that may cause differences in pain assessment between the child, parent, and nurse, as well as differences or compatibility between the parent and nurse. The children included in our study were at the appropriate age and mental and health status to express their pain appropriately using both scores. Based on the results obtained from these children who can express themselves, we will have an idea about the effectiveness of treatment given by a proxy (parent and nurse) in children who cannot express themselves and which pain score can be used to determine the pain intensity most compatible with the child in terms of proxies. Although the validity of this methodology has not been definitively established, parent and nurse assessments using the VAS and NRS are frequently used as proxy measures for pain assessment in children who are unable to self-report.^12^

## Methods

This study is a prospective randomized study conducted in the Pediatric Surgery, Orthopedics and Traumatology, Ear Nose and Throat, and Ophthalmology Operating Rooms at Ankara University Faculty of Medicine Hospitals between January and December 2022 after the approval of Ankara University Faculty of Medicine, Human Research Ethics Committee (approval no.: İ9-576-21, dated: October 14, 2024).

Children scheduled for elective minor-intermediate or major surgery between the ages of 7-12 and 12-18 years old, with the American Society of Anesthesiologists (ASA) health status level I-III and their parents were included in the study after giving written informed consent. The patient and/or parents who did not agree to participate in the study, the patient and/or the parent who has cognitive dysfunction, the child who has an ASA health status of level IV-V, liver or kidney dysfunction, receiving daily analgesic treatment due to chronic pain, and has a history of allergy to any of the medications used were excluded.

Patients, their parents, and nurses were informed before enrollment about the visual pain scale and NRS, which will be used in pain assessment. It was explained that on the visual pain scale, one end of a 100-mm line represents no pain, while the other end represents the most severe pain experienced. Measurement was performed after the assessor marked the pain on the chart. Additionally, it was explained that “0” on the NRS indicates no pain, while “10” indicates the most severe pain experienced. On the NRS, the evaluator was asked to express pain using a number between “0 and 10”. Although the nurses participating in the study were assigned to different wards, they possessed substantial knowledge and experience with both pain scales commonly utilized in surgical clinics. However, they were informed about the pain scales to be used in the study, and their consent was obtained.

All assessments were performed at the 2^nd^ postoperative hour. All three participants (child, parent, nurse) were unaware of each other, and the procedure was performed separately via VAS and NRS at the clinical ward and recorded by the same anaesthesiologist. Upon entering the child’s room to assess pain levels, the parent was respectfully requested to step outside, ensuring that pain assessments from both the child and parent could be obtained independently. Pain scores from the child and parent were recorded separately to prevent mutual influence. Additionally, the nurse conducting the assessment remained blinded to both the child’s and parent’s scores to avoid any potential bias. Pain was then systematically evaluated using standardized assessment scales, with all findings documented.

Patients were randomized according to the age of the children and the extent of surgery to be performed and were divided into four groups:

• Group 1A: children aged 7-12 years old who will undergo minor or intermediate surgery;

• Group 1B: children aged 7-12 years old who will undergo major surgery;

• Group 2A: children aged 13-18 years old who will undergo minor or intermediate surgery;

• Group 2B: children aged 13-18 who would undergo major surgery.

Factors such as the child’s gender, age, weight, height, comorbidities, use of medication, previous surgeries that could affect pain assessment, and type of planned surgical procedure were recorded preoperatively. Furthermore, the gender (mother or father), age, marital status (currently married or divorced), number of children, and education level of the parents participating in the study were questioned and recorded.

In all patients, anaesthesia and pain management were routine departmental practices and were not interfered with by the researchers. The same investigator provided all preoperative briefings, discharged the patients from the recovery unit, and visited the child, his or her parents, and the nurse for pain assessment at the 2^nd^ postoperative hour (ŞB).

Since children may perceive disturbing factors, such as intravenous lines and blood pressure cuffs, etc., as pain, it was confirmed that the body region they complained about was compatible with the surgery site before the children were asked to evaluate for pain.

### Statistical Analysis

The number of children was 172 in total, including 86 major and 86 minor-intermediate surgeries to detect an effect of 0.25 (Cohen-f) between family, nurse, and patient in terms of VAS scores after minor-intermediate or major surgical procedures in paediatric patients of different ages with a power of 0.80 at a significance level of 0.05. The sample size calculation was performed using G^*^Power software (version 3.1.9.2).

The SPSS 11.5 program was used to analyze the data. Mean ± standard deviation and median (minimum-maximum) were used as descriptive variables for quantitative variables, and the number of patients (percentage) was used for qualitative variables. The intraclass correlation coefficient (ICC) was used to examine parent, nurse, and child agreement on VAS and NRS. Univariate and multivariate linear regression analyses were performed to investigate factors affecting the child-parent VAS and NRS differences. *P *< 0.05 was considered as the statistical significance level.

## Results

**Figure 1:**
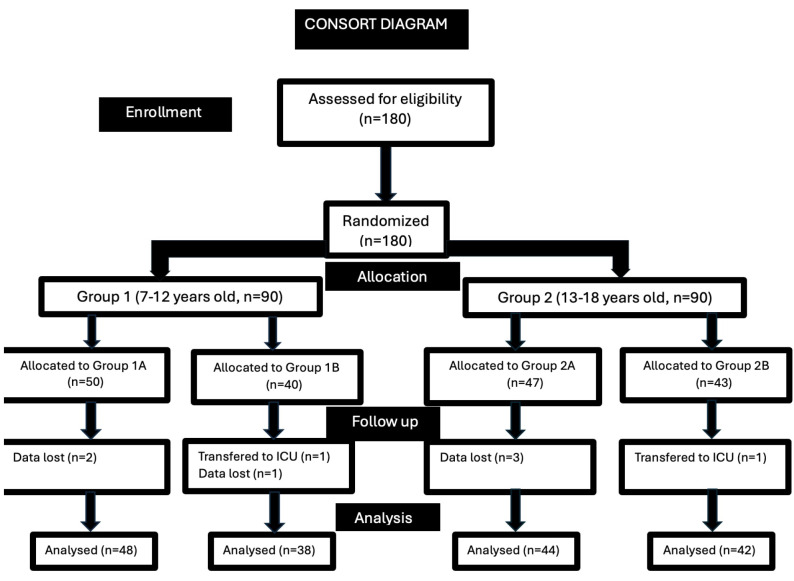
CONSORT flow diagram for the study. ICU, intensive care unit.

A total of 180 children, 90 patients in each age group (7-12 years and 13-18 years), and their parents were included in the study, taking into account data loss. Four children from each age group were excluded from the study due to insufficient data recording, and data from 172 children were analyzed (Figure 1). 27.9% of the children included in the study had minor/intermediate surgery between the ages of 7-12 years (Group 1A), 22.1% had major surgery between the ages of 7-12 (Group 1B), 25.6% had minor/intermediate surgery and surgery between the ages of 13-18 (Group 2A), and 24.4% were in the major surgery group between the ages of 13-18 years (Group 2B) (Table 1).

Values for VAS and NRS, which were reported separately by the child, parent, and nurse, are shown for all children in Table 2, regardless of the surgery grade.

The results of the ICC method were used to examine the compliance scores of the VAS scores for each study group (child-parent and child-nurse), as presented in Table 3.

When the child-parent and child-nurse harmony scores were compared, it was concluded that the parent’s VAS reporting was better than the nurse’s in Group 1A, 2A, and 2B. In Group 1B; it was concluded that the VAS reporting of the parent and nurse were similar (Table 3).

When all children were evaluated using the VAS, excellent (ICC= 0.903) agreement was found between the child and parent, and good agreement (ICC =0.852) was found between the child and nurse. When the child-parent and child-nurse harmony scores were compared, it was concluded that the parent’s VAS reporting was better than the nurse’s (Table 3).

The results of the ICC method were used to examine the compliance scores of the NRS values for each study group (child-parent and child-nurse), as shown in Table 4. When the child-parent and child-nurse harmony scores were compared, it was concluded that the parent’s NRS reporting was better than the nurse’s in all groups.

When all children were evaluated using the NRS, excellent (ICC =0.900) agreement was found between the child and parent, and good agreement (ICC =0.842) was found between the child and nurse. When the child-parent and child-nurse harmony scores were compared, it was concluded that the parent’s NRS reporting was better than that of the nurse (Table 4). When the general ICC scores for VAS and NRS scores were compared for both parents and nurses, it was seen that the VAS and NRS scores gave very similar results.

When the child-parent harmony scores for the mother and father were compared, it was concluded that the father’s VAS reporting was better than the mother’s. When the child-parent adjustment scores for the mother and father were compared, it was concluded that the father’s NRS score was slightly better than the mother’s (Table 5). The literature review revealed a notable gap, as no studies specifically examined whether mothers or fathers are more accurate in predicting a child’s pain. This finding is a significant and noteworthy aspect of our study and has the potential to make a valuable contribution to the existing literature.

There were no significant differences between the groups in terms of the gender of the patient, previous surgery, parents’ education level, marital status, or number of children they had.

In Table 6, the effect of previous surgery on pain scores was examined, and only the effect on the VAS child pain score was found to be significant (*P*=0.038). The VAS child score in patients who had previously undergone surgery was 0.961 units lower than that in patients who had not undergone surgery. Previous surgery alone explained 2.5% of the variation in the VAS score of children. For the other pain scores, patients who had undergone previous surgery had lower pain scores than those who had not previously undergone surgery; however, this difference was not significant.

## Discussion

Acute pain is a stimulating, complex, dynamic, and subjective experience. The subjective nature of pain has led to self-reporting of pain as the most effective way to measure pain. The most important step in postoperative pain management is pain assessment during follow-up. In paediatric patients, caregivers must evaluate the severity of pain and contribute to and monitor pain treatment in terms of managing pain in children, especially in children who cannot express their pain for various reasons. However, it is important to accurately measure pain, which is a subjective but important complaint, both at the beginning and during treatment, to avoid adverse effects caused by less or more treatment than needed.

The results of this study show that when children between the ages of 7-18 years old underwent minor-intermediate or major surgery, their parents could perform a closer pain assessment with their children using VAS and NRS pain scales. Nurses were able to perform less similar assessments of children’s pain than parents with both pain scores.

Similar to the results of this study, Rajasagaram et al.^8^ examined 86 children between the ages of 3 and 15 years who underwent a painful procedure and found that nurses’ scores were significantly lower than the children’s scores for each age group and parents’ scores were equal to or lower than the children’s scores. Khin Hla et al.^12^ showed that healthcare professionals tend to underestimate postoperative pain in children who undergo outpatient surgery. Our findings showed that nurses rated children’s pain lower (in other words, they underestimated it) in all groups, regardless of the children’s age, surgical grade and gender. Nurses are a vital part of patient care and treatment, especially in inpatient settings. Nurses actively participate in patient treatment, monitoring, and follow-up. Moreover, nurses usually interact more frequently with patients during hospitalization than physicians. One explanation for this may be that the parent takes his/her own child’s usual behavior as his/her frame of reference, while the nurse has extensive experience with other children who have undergone surgery. Another reason may be that nurse with different degrees of expertise performed the evaluation, as in our study. Lack of knowledge or workload among nurses may have affected the evaluations. Our study was conducted with nurses working in the surgical department of a university hospital, mostly in paediatric departments. Furthermore, despite the fact that all nurses were informed about the pain scales that were planned to be used before starting the study, they underestimated the children’s pain with both pain scores. Insufficient knowledge about pain assessment despite pre-study information may also be a reason, but children and parents gave similar scores with similar information. A more likely cause may be the difference in experience levels and workload of nurses because they were randomly selected because they were working in shift patterns. In fact, nurses can take into account parameters that can be affected by pain, such as concurrent heart rate, oxygen saturation, blood pressure, body posture, and facial expressions, albeit unintentionally (which is necessary for patient follow-up and treatment). Increasing the level of knowledge and awareness of nurses and healthcare workers about pain, pain measurement, and treatment will make a very important contribution to pain management in all patient groups, especially those who cannot report their pain levels, such as paediatric patients. Furthermore, if healthcare providers keep themselves up-to-date on pain, they will be able to provide information to patients and their families on this matter.

The VAS and NRS pain scores used together in this study showed that children rated their pain at a similar intensity. Additionally, when the general compliance scores of the parents and nurses who assessed the child’s pain were compared, very similar results were obtained with the two scores. For this reason, we believe that VAS or NRS can be used to evaluate pain in children between the ages of 7-18 years effectively and reliably.

In our study, it was observed that as the age of children increased in minor-intermediate surgeries, the compatibility between the child and parent decreased in both the VAS and NRS scores. Knutsson et al.^14^ also reached a conclusion similar to our study. When they questioned the pain of children aged between 3 and 10 years who underwent adenoidectomy, a surgery that can be considered minor intermediate, using the VAS score, they concluded that harmony with the parents decreased as the age of the children increased.^14^

In children who underwent major surgery, we found that as the age of the child increased, the compatibility between the child and parent increased in terms of both the VAS and NRS scores. The reason for the increase in compliance with major surgeries with age may be that families made a closer prediction of their children by scoring them higher than normal due to their perception that major surgeries would be more painful. In our literature review, we did not find any data showing the compatibility between age and the age of the child and parent during major surgeries. For this reason, it is possible to consider it an important result of our study.

In a study by Kaminsky et al.^17^ which included children between the ages of 2 and 15 years who underwent adenoidectomy and/or tonsillectomy, parents estimated their children’s postoperative pain to be higher than their children. However, the amount of analgesic administered did not exceed the safe limits and was not considered unnecessary. The fact that Kaminsky et al.’s^17^ study was conducted in different hospitals, used pain scores that were different from our study, and evaluated a 3-day pain score and analgesic treatment may have caused differences in our results.

When we evaluated the effect of whether the parent was a mother or father on the ICC values of the child’s pain using VAS and NRS, we found that fathers were better at estimating their children’s pain than mothers. Although the mothers were compliant, they scored lower. The lower number of fathers participating in our study (19.2% vs. 80.8%) may be due to the differences in mother-child and father-child relationships in society. No study has been found in the literature on which mother and father predict the child’s pain more closely. This result is also one of the most remarkable and important points of our study.

In a study of 102 patients conducted by Logan and Rose^18^ adolescent girls reported higher rates of “lowest daily pain” and “average daily pain” than adolescent boys after an overnight hospital stay after surgery. In this study, no significant difference was found in terms of sex.

Another significant finding of our study was that 44.8% of the children had previously undergone surgery. The elevated scores on the VAS and NRS for pain observed in these children, regardless of age group or type of surgery, could be attributed to heightened anxiety and apprehension about the potential adverse outcomes associated with earlier surgical interventions. This suggests that prior surgical experiences may significantly influence pain perception and anxiety in children, highlighting the importance of comprehensive preoperative psychological assessment and tailored postoperative care strategies to mitigate these concerns.

It is expected that our study will inform more accurate assessments of postoperative pain and serve as a foundation for future research, particularly in paediatric patients undergoing the same type of surgery. Additionally, providing education to both children and their parents about the use of pain scales before surgery could improve the validity of pain evaluations and enhance the overall understanding of pain management in paediatric surgical populations.

### Study Limitations

As with the limitations of postoperative pain assessments, our study has several limitations. First, we performed the evaluation only once postoperatively. The results of evaluations performed at different postoperative periods may differ. Another limitation is that not knowing the baseline psychological state of the parent conducting the pain assessment, how much time they spend with their children in their normal social life, and their relationship characteristics may have affected their evaluation. Finally, nurses with different levels of experience in various surgical clinics evaluated the patients.

## Conclusion

The results of this study showed that two hours after surgery, parents were better at approximating their child’s pain level than nurses in children aged 7-18 years old who could easily express themselves based on the VAS and NRS. When a child cannot express their pain, parents can use both pain scales to measure pain effectively and reliably. These results also show that parents’ assessments can be used during post-discharge follow-up and the regulation of children’s pain management at home. In our study, nurses underestimated the child’s pain for both pain scores. Clearly, there is a need for continuing education and awareness among nurses and other healthcare professionals regarding pain, pain measurement, and treatment. There is a need for studies to determine the appropriate pain scale in order to make an accurate assessment for children and as a proxy to be used in the initiation and follow-up of pain treatment in different age and patient groups.

## Ethics

**Ethics Committee Approval:** Ethics committee approval was received from the Ankara University Faculty of Medicine, Human Research Ethics Committee (approval no.: İ9-576-21, dated: October 14, 2024) before the study execution.

**Informed Consent:** Written informed consent was obtained from all participants.

## Figures and Tables

**Table 1. Basic Patient and Parent Characteristics:** 

**Variables**		
**Group, n (%)**	1A	48 (27.9%)
	1B	38 (22.1%)
	2A	44 (25.6%)
	2B	42 (24.4%)
**Age (years)**	Mean ± SD	11.8±3.5
	Median (Min.-Max.)	12.0 (7.0-18.0)
**Sex, n (%)**	Girl	53 (30.8%)
	Boy	119 (69.2%)
**Operation** **Grade, n (%)**	Minor/intermediate surgery	92 (53.5%)
	Major surgery	80 (46.5%)
**Operation history, n (%)**	No	95 (55.2%)
	Yes	77 (44.8%)
**Parents, n (%)**	Mother	139 (80.8%)
	Father	33 (19.2%)
**Parental educational status, n (%)**	Primary	65 (37.7%)
	High school	51 (29.7%)
	University	56 (32.6%)

Mean, average; SD, standard deviation; Min., minimum; Max., maximum.

**Table 2. VAS and NRS Scores:** 

-	**95% CI (Lower-Upper limit)**	**Median (Min.-Max.)**
**Children VAS**	3.94-4.85 (*P *< 0.001)	4.5 (0.0-10.0)
**Parent VAS**	3.77-4.64 (*P *< 0.001)	4.0 (0.0-10.0)
**Nurse VAS**	2.58-3.29 (*P *< 0.001)	3.0 (0.0-8.0)
**Children NRS**	4.46-5.41 (*P *< 0.001)	5.0 (0.0-10.0)
**Parent NRS**	3.88-4.57 (*P *< 0.001)	4.0 (0.0-10.0)
**Nurse NRS**	2.58-3.29 (*P *< 0.001)	3.0 (0.0-8.0)

CI, confidence interval; Min., minimum; Max., maximum; VAS, visual analogue scale; NRS, numerical rating scale.

**Table 3. ICC Values for the VAS Study Groups:** 

**Groups**	**Child-Parent**			**Child-Nurse**		
	**ICC**	**95% CI (Lower-Upper limit)**	***P* value**	**ICC**	**95% CI (Lower-Upper limit)**	***P* value**
**1A**	0.903	0.833-0.944	<0.001	0.804	0.676-0.885	<0.001
**1B**	0.802	0.651-0.892	<0.001	0.804	0.654-0.893	<0.001
**2A**	0.860	0.758-0.921	<0.001	0.766	0.609-0.865	<0.001
**2B**	0.885	0.797-0.937	<0.001	0.807	0.668-0.891	<0.001
**All patients**	0.903	0.871-0.927	<0.001	0.852	0.805-0.888	<0.001

CI, confidence interval; ICC, intraclass correlation coefficient; VAS, visual analogue scale.

**Table 4. ICC Values of the Study Groups for NRS:** 

**Groups**	**Child-Parent**			**Child-Nurse**		
	**ICC**	**95% CI (Lower-Upper Limit)**	***P* value**	**ICC**	**95% CI (Lower-Upper Limit)**	***P* value**
**1A**	0.895	0.820-0.940	<0.001	0.808	0.682-0.888	<0.001
**1B**	0.813	0.669-0.898	<0.001	0.744	0.559-0.858	<0.001
**2A**	0.883	0.795-0.934	<0.001	0.768	0.612-0.866	<0.001
**2B**	0.838	0.718-0.909	<0.001	0.784	0.633-0.878	<0.001
**All patients**	0.900	0.867-0.925	<0.001	0.842	0.792-0.880	<0.001

CI, confidence interval; ICC, intraclass correlation coefficient; NRS, numerical rating scale.

**Table 5. Parental ICC Values for VAS and NRS Scores:** 

**Measurement**	**Parent**	**Child-Parent**		
		**ICC**	**95% CI (Lower-Upper limit)**	***P* value**
**VAS**	**Mother**	0.898	0.860-0.926	<0.001
	**Father**	0.935	0.872-0.967	<0.001
**NRS**	**Mother**	0.899	0.861-0.927	<0.001
	**Father**	0.905	0.816-0.952	<0.001

CI, confidence interval; ICC, intraclass correlation coefficient; NRS, numerical rating scale; VAS: visual analogue scale.

**Table 6. Effect of Previous Surgery on Pain Score:** 

**Variables**	**β**	**SE**	***P *value**	**R^2^**	**95% CI**
**VAS Child**	-0.961	0.460	0.038	0.025	-1,869-0.054
**VAS Parent**	-0.755	0.445	0.092	0.017	-1,634-0.123
**VAS Nurse**	-0.445	0.366	0.225	0.009	-1,167-0.277
**NRS Child**	-0.765	0.482	0.115	0.015	-1,716-0.187
**NRS Parent**	-0.750	0.451	0.098	0.016	-1,641-0.140
**NRS Nurse**	-0.389	0.365	0.289	0.007	-1,110-0.333

CI, confidence interval; VAS, visual analogue scale; NRS, numerical rating scale; SE, standard error.
